# Maxillary sinus membrane perforation rate utilizing osseodensification‐mediated transcrestal sinus floor elevation: A multicenter clinical study

**DOI:** 10.1111/cid.13368

**Published:** 2024-08-26

**Authors:** Ziv Mazor, Joao Gaspar, Robert Silva, Snjezana Pohl, Yazad Gandhi, Salah Huwais, Edmara Tatiely Pedroso Bergamo, Estevam Augusto Bonfante, Rodrigo Neiva

**Affiliations:** ^1^ Private Practice Tel Aviv Israel; ^2^ Department of Oral Surgery Egas Moniz School of Health and Science Caparica Portugal; ^3^ Private Practice Implanteperio Institute São Paulo Brazil; ^4^ Department of Oral Medicine and Periodontology University of Rijeka, Private Clinic Rident Rijeka Croatia; ^5^ Oral Surgery and Implantology Private Practice Mumbai India; ^6^ Saifee Hospital Mumbai India; ^7^ Department of Periodontics University of Pennsylvania, School of Dental Medicine Philadelphia Pennsylvania USA; ^8^ Department of Prosthodontics and Periodontology University of São Paulo – Bauru School of Dentistry Bauru Brazil

**Keywords:** dental implants, membrane perforation, osseodensification, osseointegration, sinus floor elevation

## Abstract

**Purpose:**

This multicenter cross‐sectional clinical study aimed to evaluate the membrane perforation rate during transcrestal sinus floor elevation (TSFE) using osseodensification (OD) burs and assess risk factors associated with the procedure.

**Materials and Methods:**

This study was conducted in six centers, following ethical standards and approved by local committees. It included patients over 18 years old missing maxillary posterior teeth with crestal residual bone height (RBH) ≥2 and ≤6 mm. Preoperative evaluations were done, including CBCT scans, to assess bone dimensions and sinus health. All centers and surgeons followed a standardized surgical protocol for TSFE using OD burs. Surgical complications, particularly sinus membrane perforations, were recorded and analyzed. Factors such as implant site, premolars or molars, as well as, healed or fresh socket, along with initial RBH were evaluated for their impact on membrane perforation rate. Descriptive statistics, *χ* ^2^, and logistic regression analysis were used to analyze the data.

**Results:**

A total of 621 subjects with an average age of 57.9 years were included. Sinus lifting was performed at 670 sites, with 621 implants placed in the maxilla. The majority of sinus lifts were done in the molar region (76.87%) and in healed bone sites (74.33%). The average RBH was 5.1 mm (ranging from 2 to 7 mm). Sinus membrane perforation occurred in 49 cases (7.31%). RBH ≤3 mm posed a risk factor for sinus membrane perforations followed by RBH >3 and ≤5 mm. Tooth region and implant site were not associated as risk factors for sinus membrane perforation.

**Conclusion:**

OD drilling used for TSFE resulted in low membrane perforation rate. Challenging scenarios of severe posterior maxillary atrophy presented as risk factors for increased perforation rate.

**Summary box**

**What is known**

- Clinical studies showed that the osseodensification technique outperformed the lateral window technique for sinus floor elevation in providing higher implant insertion torque levels, implant primary stability, and in several patient‐reported outcome measures.
- Most studies have been conducted in a single center and involved a limited number of subjects.

**What this study adds**

- This is the first multicenter clinical study that evaluated the sinus membrane perforation rate using the osseodensification technique on a robust sample size of 621 patients.
- The membrane perforation rate was low, and risk factors associated with this outcome have been identified.

## 1 INTRODUCTION

After tooth loss, the posterior maxilla undergoes significant volume changes.^1^ Changes in alveolar bone height and width may occur due to a concurrent alveolar ridge resorption and sinus pneumatization, which may increase with age and tooth loss.^2^, ^3^ Implant supported rehabilitation in the posterior maxilla is critical due to the fact that this location is associated with the highest implant failure rate in the long‐term.^4^ Therefore, there is more need to introduce and validate maxillary sinus augmentation treatment modalities to increase bone volume and subsequent implant success rate in these areas.

Several techniques have been proposed to address the significant loss of native bone in the posterior region of the maxilla. These techniques include sinus augmentation procedures prior to or simultaneously with implant placement, which help to increase the height and volume of sinus floor.^5^ The elevation of the Schneiderian membrane can be achieved via surgical access through the lateral maxillary wall (lateral window technique)^5^ or transcrestal sinus floor elevation (TSFE) utilizing osteotomes, which has been proposed as a less invasive approach by Tatum and Summers.^5^, ^6^ However, the most commonly reported complication in both surgical protocols is the perforation of the sinus membrane (7%–58%).^7^, ^8^ An intact sinus membrane is needed to maintain the osteogenic space and prevent subsequent infection.^9^, ^10^, ^11^ Membrane perforation may lead to other complications, including migration of the graft into the sinus, sinusitis, infection, graft loss, and ultimately failure of the surgical procedure.^12^ Sinus perforations can often times go undetected and several techniques have been proposed to evaluate membrane integrity, such as Valsalva maneuver,^13^ the nose‐blow test,^14^ the mirror fog up test, or direct visualization among others.^15^

Several factors have been proposed as risk factors for membrane perforation during sinus lifting procedures. These may include Schneiderian membrane thickness, residual bone height (RBH), sinus width, the surgical technique and operator experience, presence of septa, bony wall thickness, and smoking.^16^, ^17^ Anatomical factors and surgical limitations have been suggested to impact the predictability of the sinus grafting procedures and the overall risk for membrane perforation.^18^ Traditional transcrestal sinus grafting methods are associated in the literature with lower risk of sinus membrane perforation, but a minimum of 5‐mm RBH is required to reduce such a risk.^6^, ^19^, ^20^ A significant statistical correlation exists between initial RBH and membrane perforation due to technical and surgical difficulties.^21^, ^22^

Among the factors mentioned above, the thickness of the sinus membrane has been considered as the main factor to influence membrane perforation. It has been reported that the average thickness of membrane to be 1 mm in healthy patients.^23^ Age shows a strong correlation with membrane thickness, whereas sex and seasonal time have not shown significant effects.^24^ Three‐dimensional imaging technologies measurements tend to overestimate membrane thickness by 2.5 times when compared with histological analysis. Conditions such as periodontitis and smoking can also contribute to increase membrane thickness and reduce its ability to stretch.^23^ In fact, maxillary sinus membrane mechanical properties and its ability to stretch play a crucial role in the crestal sinus grafting procedure. It has been reported that the Schneiderian membrane can stretch up to 132% of its original size in one dimension, and up to 125% in two dimensions.^25^ Despite that, the membrane may get perforated if the local tension exceeds its stretching ability,^26^ which is directly related to several anatomical and procedural variations.

In this context, osseodensification (OD) is a non‐excavating universal additive osteotomy technique that utilizes specially designed burs to expand a pilot osteotomy in trabecular bone and autograft bone into the adjacent trabecular space structure both laterally and apically.^27^ When OD burs are used in a non‐cutting direction (counterclockwise‐CCW) with adequate irrigation, they create a hydraulic wave ahead of the point of contact that compacts and autografts bone both apically and laterally into the trabecular space.^27^, ^28^, ^29^, ^30^, ^31^, ^32^, ^33^, ^34^, ^35^ According to a recent clinical study^36^ which compared the onset of vascular bleeding and the osteotomy blood fill between OD and conventional drilling, OD did not seem to negatively affect or induce loss of bone vascularity. An in vivo study in low density sheep bone has shown that significant gains in implant primary stability can be achieved using OD drilling, compared with conventional drilling using drills of identical geometry, and that bone chip residues are autografted into the trabecular space resulting in a higher bone to implant contact at different follow‐up evaluations.^29^ Also, regardless of implant surface treatment (machined vs. acid‐etched), higher implant insertion torque has been reported for OD compared with subtractive regular drilling in low density bone; OD has also resulted in high amounts of bone to implant contact and Bone Area Fraction Occupancy in both machined‐surface and rough‐surface implants with no statistically significant difference between them.^28^ A multicenter prospective clinical study has demonstrated that with several implant designs, and regardless of anatomical site, not only higher insertion torque values were observed with OD compared with conventional drilling, but of utmost importance was the fact that implant stability quotients values were sustained and increased over time, whereas implant stability quotients values in conventional drilling decreased significantly at 3 weeks.^37^ This was also confirmed in a multicenter retrospective study with 5 years follow up.^38^

Additionally, when the OD drilling protocol is used close to the sinus floor, the resultant apical hydraulic compaction wave created with the autogenous bone slurry produces a controlled pressure upon the Schneiderian membrane, which in turn is elevated along with autografts that are introduced between the membrane and the sinus floor.^39^ Contrary to the Summers osteotome technique, which is limited to cases where RBH is ≥5 mm,^6^ the OD sinus grafting protocol is reported with adequate success in several clinical prospective and long‐term retrospective clinical studies, with RBH as low as 2 mm with vertical increase post‐grafting of up to 10 mm. This has been reported in a multicenter study, with minimum sinus membrane perforations and resulting in a 97% cumulative implant survival rate after an up to 64 months follow‐up.^39^ Even in cases of severe atrophy of the posterior maxilla (RBH ≥2 and <6 mm), OD protocol has shown to effectively improve implant stability and elevate the sinus floor with no membrane perforation, confirmed by postoperative cone‐beam computed tomography scans, in 17 patients.^40^

From a patient perspective, measured through patient‐reported outcome measures, sinus floor elevation (SFE) with lateral window technique has shown inferior outcomes in pain experience, surgery duration, surgical complications, self‐perceived quality of life, postoperative edema, and analgesics intake when compared with the OD sinus grafting as reported in a randomized clinical trial involving 20 patients.^41^ Although these promising results might favor implant placement in the atrophic posterior maxilla, avoiding more invasive sinus lifting approaches, there is still risk for membrane perforation, requiring further investigations in larger sample sizes and in a multicenter scenario. Hence, this multicenter clinical study aimed to evaluate the SFE via a transcrestal approach using OD burs and assess membrane perforation rate, along with risk factors associated with the procedure. The postulated alternative hypothesis was that OD technique used for TSFE would result in low membrane perforation rate.

## 2 MATERIALS AND METHODS

This multicenter cross‐sectional study was carried out in accordance with the ethical standards outlined in 1964 Declaration of Helsinki. The study was performed in six different centers and approved by their respective local ethical committees (protocol ID ODSINUSPERF), and according to STROBE guidelines. Registration was made at Clinical Trials (clinicaltrials.gov) under the ID # NCT05954273.

Patients in need to receive dental implants in posterior maxillary edentulous spaces were included. All patients were subjected to a preliminary evaluation that included careful review of their medical and dental histories, detailed clinical examination, and evaluation of oral hygiene. The inclusion criteria were patients of at least 18 years of age missing maxillary posterior teeth with ≥2 to ≤6 mm of RBH between the crest of the ridge and the sinus floor, and maxillary alveolar ridge width ≥4 mm. The exclusion criteria were patients with (a) >80 years of age and (b) presence of sinus pathologies (as detected on cone beam computed tomography [CBCT] scan), presence of septa in line with the proposed osteotomy site, systemic disorders (uncontrolled diabetes mellitus, bleeding disorders, compromised immune system, irradiated patients, treatment with steroids or bisphosphonates), alcoholism, excessive smoking (>20 cigarettes per day),^42^ and tobacco chewing habit.

### 2.1 Preoperative evaluation

All patients had (CBCT) scans prior to implant placement for surgical planning and assessment of bone dimensions around the implantation site. Careful assessment of the sinus health and anatomy was made on all sections of the CBCT scans to evaluate the sinus floor and rule out any membrane thickening or allied sinus pathology. A detailed history was obtained to rule out any evidence of sinus disease or prior surgical intervention. Clinical assessment was carried out for every case to rule out any evidence of sinus disease. Each patient received detailed description of the study protocol, signed the inform consent form and gave written approval to be included in the study population.

The following implant systems were used: tapered screw‐vent implants (ZimVie, Palm Beach, FL, USA), tapered implants (Biohorizons, Birmingham, AL, USA), Anyridge implants (Megagen, Fairlawn, NJ, USA), Nobel Active implants (Nobel Biocare, Brea, CA, USA), ID^CAM^ ST (Implant Diffusion International, Montreuil, France), and Bego Semandos RSX (Bego, Bremen, Germany). The implants placed in the current study were usually: regular (≥3.75 to <5 mm) and wide (≥5 mm) diameters, as well as regular (≥10 to <12 mm) and long (≥12 mm) lengths.

### 2.2 Surgical procedure

All centers and surgeons followed a standardized surgical protocol. The operative procedure was carried out under local anesthesia administered by way of a posterior superior alveolar, and greater palatine with occasional infra‐orbital nerve block specially in premolar site placement. A full thickness mucoperiosteal flap was reflected, using either a crestal or palatal horizontal incision with vertical release incision when and if needed to expose the surgical site.

**FIGURE 1:**
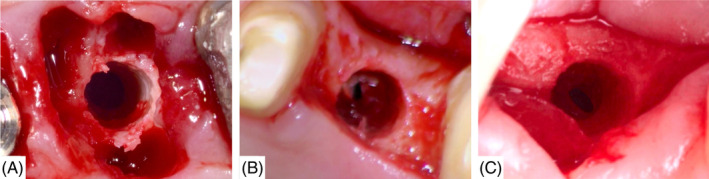
(A–C) Clinical examples of sinus membrane perforation.

All operators have followed OD crestal sinus graft protocols published by the company (www.versah.com). In brief, in cases with RBH >5 mm, and after full flap reflection, the pilot drill was used to start the osteotomy at the desired site at 1100 rpm, and the drill was stopped 1‐mm below the sinus floor. Thereafter, an OD bur (Densah bur VT1525, Versah, Michigan, Jackson, USA) was used in 1100 rpm in a CCW direction with copious irrigation to reach the sinus floor. After that, the following OD bur (Densah bur VT2535, Versah) was used in 1100 rpm in a CCW direction with copious irrigation and vertical bouncing motion to enter the sinus up to 3 mm, lifting the membrane and depositing up to 3 mm of autogenous bone below the membrane. Direct visual membrane integrity verification, using magnification, in every preparation step was done with subsequent registration of any perforation (Figure 1). Subsequently, wider OD burs were used in the same operation mode to increase osteotomy diameters and autograft the lifted space. Final osteotomy diameter was performed according to the planned implant diameter.

Same clinical protocol was followed in cases with RBH of 4–5 mm, except for the use of the pilot drill and the use of additional bone graft materials to lift the sinus membrane beyond the 3 mm initial lift provided by the autogenous bone grafting. In this scenario, bone grafting materials were slowly propelled after final osteotomy width preparation using the final OD bur at 100–200 rpm with no irrigation to secure additional vertical lifting and grafting beyond the initial 3 mm lift, and further to the planned implant length level (Versah Sinus grafting protocol II, www.versah.com).

In cases with RBH ≤3 mm, the pilot drill and first OD Bur (Densah bur VT1525) were skipped. The initial site preparation started with the OD bur (Densah bur VT2535, Versah) at 1100 rpm in a CCW direction with copious irrigation and vertical bouncing motion to reach the sinus floor. Then, the wider OD bur (Densah bur VT3545, Versah), in the same operation mode, was used to enter the sinus cavity, lifting the membrane, and depositing up to 3 mm of autogenous bone below the membrane. Subsequently, the following OD bur (Densah bur VT4555, Versah) was used to increase osteotomy diameter and further lift the membrane, depositing additional autografting. After the final osteotomy diameter was created, bone grafting materials were slowly propelled into the lifted space using the final OD bur at 100–200 rpm with no irrigation to secure additional vertical lifting and grafting beyond the initial 3 mm lift, and further to the planned implant length level. Different bone grafting materials were used according to each operator clinical judgment.

The same protocols were followed in both fresh and healed sockets as long as the ridge‐width minimum was met. If fresh socket apical width did not meet the minimum width needed for a specific protocol, the sinus grafting was not done at the day of extraction, and the socket was grafted for a later second stage sinus lift post‐healing. Bone height or the amount of bone below the sinus floor dictated which protocol should be utilized (which Densah Bur to enter the sinus with). For instance, a 5 mm wide healed ridge with 4–5 mm of bone below the sinus floor received the same protocol as for an extraction socket with 5 mm width and 4–5 mm of bone below the sinus floor (protocol II). If the amount of bone height at the apex of an extraction socket was 2–3 mm, it dictated the need for 7 mm apical ridge width and it followed the same protocol of healed ridge with 2–3 mm below the sinus floor (protocol III).

After sinus grafting osteotomy, all cases received simultaneous implant placement except in cases with signs of membrane perforation. All incidents of membrane perforation were diagnosed by direct observation with loupes magnification and recorded. Perforation cases were treated by grafting the osteotomy without allowing the grafting materials into the sinus cavity and achieve full flap closure. Post‐implant placement radiographs were taken to confirm the lack of radiographic evidence of biomaterials propelling into the sinus and to rule out any perforation of the graft materials into the sinus cavity.

Patients were instructed to follow a soft and cold diet in the first 3 days after surgery, along with instructions for oral hygiene. Additional prescription included antibiotics coverage for at least 1‐week post‐surgery with anti‐inflammatory and analgesic medications for 3 days.

### 2.3 Parameters investigated

Surgical complications, particularly sinus membrane perforations, were recorded. Some factors were evaluated to assess their influence on the membrane perforation rate, including:

- Site for implant placement, which was categorized as: (i) molar and (ii) premolar sites, as well as (i) immediate and (ii) healed sites.
- Initial RBH: bone dimensions were analyzed pre‐operatively using CBCT. Then, the initial crestal bone height of each implant site was categorized as: (i) ≤3 mm, (ii) between 3 and 5 mm, and (iii) >5 mm.

Descriptive statistics of numerical variables, such as patients' data along with bone dimensions and implant distribution were expressed as mean values and the corresponding 95% confidence interval or standard deviation. Categorical variables were expressed as number and percentages. Sinus membrane perforation was tested for an association with the corresponding risk factors (initial crestal bone height and implant site) using the *χ* ^2^ test. Logistic regression analysis was used to model the odds ratio (OR) of sinus membrane perforation by the corresponding risk factors. The analyses were accomplished using SPSS with a significance level of 5% (IBM SPSS 23, IBM Corp., Armonk, NY).

## 3 RESULTS

A total of 621 subjects, with a mean age of 57.9 ± 1.05 years, were included in this study. Overall, the clinical findings demonstrated uneventful sinus lifting and implant surgery procedures, with no excessive bleeding, peri‐implant soft tissue complications such as suture loosening, inflammation, infection, or other complications.

Six‐hundred and seventy (670) sinus grafting sites were performed in six centers utilizing standardized instrumentation protocols with simultaneously 621 implants placed in the posterior region of the maxilla (Table 1). All implants placed on the same day of the sinus grafting were restored and followed in routine‐maintenance recall.

**TABLE 1:** Data distribution per center.

Center	Patient#	Sinus graft sites	Molar sites	Premolar Sites	Perforation	Perforation rate %	Implants placed	Graft material
C1	97	117	74	43	11	9.40	107	Allograft
C2	35	35	35	0	3	8.57	32	Allograft
C3	67	78	69	9	6	7.69	71	Synthetic
C4	120	111	84	27	9	8.11	110	Synthetic/xenograft
C5	98	115	86	29	8	6.95	100	Synthetic
C6	204	214	167	47	12	5.61	201	Allograft/synthetic
**Total**	**621**	**670**	**515**	**155**	**49**	**7.31**	**621**	

*Note*: Allograft FDBA (combination of cancellous/cortical) and combination of (FDBA and DFDBA), Synthetic Putty of calcium phosphosilicate combined with a polyethylene glycol and glycerine binder, and Xenograft (small particles Bovine).

Of the 670 sinus lifts, 515 were in the molar region and 155 were in the premolar region. Also, 498 sinus grafting were done in healed bone sites, whereas 172 sinus grafting were done in fresh socket sites.

The average RBH was 5.1 mm (±1.96 mm), with a range of 2–7 mm. A total of 165 sites had RBH ≤3 mm, 256 sites had RBH between 3 and 5 mm, and 249 sites had RBH >5 mm (Table 2). In 249 sites, no additional bone grafting was required, whereas in 421 sites, bone grafting was used to additionally augment the sinus prior to implant placement.

**TABLE 2:** Sinus sites according to initial crestal ridge height.

Sinus graft sites	Crestal bone height		
	>5 mm	3 < *x* ≤ 5	≤3 mm
670	249	256	165

A total of 49 (7.31%) sites had sinus perforations occurred and confirmed by direct visualization (Table 1; Figure 1).

The type of implant placement whether healed site (type IV) or immediate placement (type I) did not affect the prevalence of sinus membrane perforation rate statistically (*χ* ^2^, *p* = 0.058). In contrast, RBH <5 mm, and the molar region, were associated with a statistically significant increase in the prevalence of sinus membrane perforation rate (*χ* ^2^, *p* < 0.012; Table 3).

**TABLE 3:** Prevalence of sinus membrane perforation.

Variables	Perforation		*p*‐Value
	No	Yes	
Tooth site			
Molar	91.5%	8.5%	0.026
Premolar	96.8%	3.2%	
Implant site			
Healed	91.6%	8.4%	0.058
Fresh socket	95.9%	4.1%	
Crestal bone height			
≤3 mm	84.2%	14.2%	<0.001
>3 and <5 mm	92.6%	7.6%	
≥5 mm	98.4%	1.6%	

In the logistic regression analysis of the sinus membrane perforation, a RBH lower than 3 mm (OR = 10.130; *p* < 0.001) and between 3 and 5 mm (OR = 3.726; *p* = 0.022) was identified as significant risk factors for membrane perforation (Table 4). Tooth region, premolar and molar, and implant site, healed and fresh socket, were not associated as risk factors for sinus membrane perforation.

**TABLE 4:** Risk factors for sinus membrane perforation.

Risk factor	*B*	SE	Sig.	Odds ratio (OR)	95% Lower CI OR	95% Upper CI OR
Tooth (PM − M)	0.387	0.503	0.442	1.472	0.549	3.946
Implant site (socket − healed)	−0.667	0.441	0.130	0.513	0.216	1.218
Crest bone height (>5 mm)				1		
Crest bone height (≤3 mm)	2.315	0.565	0.0001	10.130	3.349	30.644
Crest bone height (3 < *x* ≤ 5 mm)	1.315	0.576	0.022	3.726	1.205	11.521

Abbreviations: M, molar; PM, premolar.

## 4 DISCUSSION

The present multicenter clinical study evaluated the Schneiderian membrane perforation rate, along with risk factors associated with SFE using a transcrestal approach with OD instrumentation. The postulated alternative hypothesis that OD technique used for transcrestal SFE would result in low membrane perforation rate was accepted. The overall membrane perforation in 670 sites in 621 patients across the six study centers was 7.31%. Moreover, the logistic regression analysis showed that more challenging scenarios of posterior maxillary atrophy, including RBH <3 mm (OR = 10.13) and between 3 to 5 mm (OR = 3.72), were associated with higher risk for membrane perforation.

Sinus membrane elevation via OD has been widely studied and researched as a predictable approach to increase the vertical dimension in the posterior maxilla.^39^, ^40^, ^41^, ^43^, ^44^ The Schneiderian membrane is known to vary in thickness and tenacity with little correlation to age and sex but until now the most common intra‐operative complication considered is membrane perforation.^24^, ^25^, ^26^, ^45^ Between the two main approaches, the transcrestal technique initially described by Summers,^6^ is widely accepted in clinical practice due to its minimally invasive nature.^6^ However, a systematic review has recently shown that the OD instrumentation outperforms the osteotome technique in successfully achieving higher implant primary stability following sinus membrane elevation which eventually allows for safer and earlier prosthetic reconstruction.^46^

A previous study has reported a cumulative survival rate of 90% after a follow‐up of 12 years for implants placed in the posterior maxilla in conjunction with SFE using the conventional osteotome technique.^47^ The RBH was considered as one of the most important determinants for the favorable implant survival outcome. In this context, reduced implant survival rates, ranging from 73.3% to 85.7%, have been reported when RBH was lower than 4 mm.^19^, ^22^ Thus, literature points toward a reduced implant survival rate utilizing osteotome technique in scenarios of posterior maxillary atrophy.^16^

When OD instrumentation was associated with SFE using the transcrestal approach, the reported implant survival rate was 97% for reduced crestal bone heights ranging from 3.5 to 7.3 mm.^39^ This method utilizes the plasticity of bone to its advantage by introducing specially designed burs into the osteotomy in a “bouncing” motion, in and out of the osteotomy. This movement induces a hydrodynamic compression wave ahead of the point of contact of the OD bur tip, forcing the irrigating fluid into the osteotomy and compacting the autograft particles (derived from the osteotomy walls) in an apical direction.^27^, ^35^, ^48^ This autogenous bone slurry produces a controlled pressure upon the Schneiderian membrane, which in turn is elevated along with autograft that is introduced between the membrane and the sinus floor.^39^

In the present study, it was notable that the membrane perforation rate increased and was directly related to a reduced RBH in the posterior region of the maxilla. This trend corroborates with previous literature findings that reported an increased rate of membrane perforation as a function of a reduction of RBH using both transcrestal and lateral window techniques.^17^, ^19^, ^22^ However, it is important to mention that prospective and retrospective clinical studies have reported higher implant survival rates with reduced incidence of sinus perforation when utilizing OD protocols in cases RBH ranging between 2 to 8 mm.^39^, ^40^, ^41^ A prospective clinical study has reported four times higher membrane perforation rate for sinus membrane elevation with the lateral window approach relative to OD transcrestal approach.^41^ Therefore, for scenarios with reduced RBH, OD instrumentation might be considered a more favorable option due to reduced invasiveness and risk for complications.

The association of OD burs with lateral window sinus lifting technique has been recently investigated in a goat model.^49^ The average bone thickness reported in the experiment was 1.38 ± 0.48 mm which was mainly cortical bone, and below the threshold of 2–3 mm of alveolar bone needed to safely provide adequate sinus floor lifting via OD technique. Despite the lack of trabecular bone needed to safely lift the membrane and the thin cortical lateral wall, the authors reported perforations rate of 16%, in addition to 8% “pinhole” perforations. In the reported goat study, using the OD burs to gain access to the maxillary sinus through the lateral wall failed to provide all elements needed for OD and the adequate bone structure, which may explain the increased occurrence of perforations in their experiment model.^49^, ^50^

In this study, important clinical findings were observed including the use of smaller incremental bur jumps resulting in an increased incidence of membrane perforation since the bone slurry created becomes insignificant to provide the adequate lift of the Schneiderian membrane ahead of the OD bur tip. The trabecular alveolar ridge with its collagen content is also key element to provide the adequate plasticity along with the adequate irrigation to separate the Schneiderian membrane off the sinus floor. Therefore, the need to reflect full flap to secure adequate irrigation and to utilize larger diameter instrumentation jumps in the sequence of OD burs to allow for more efficient hydraulics was noted as it may introduce more fluids and shave more bone off the osteotomies walls to push it in an apical direction, which may exert gentle vertical force on the membrane. Future studies are warranted to corroborate these clinical findings.

Although the Valsalva maneuver is the most commonly reported method used to evaluate Scheneidarian membrane perforation, its diagnostic accuracy is not known, and it is reasonable to speculate that the incidence of perforations may have been underestimated as a result of possible false negatives.^13^ Other diagnostic approaches have also been reported such as the Valsalva maneuver in combination with tactile feeling of membrane elasticity, the nose‐blow test,^14^ the mirror fog up test,^15^ among others. In the present study, high magnification with diligent adequate suction were used to confirm or rule out any perforation, and regardless of size even a pinhole perforation was reported as a perforation. If perforation was detected, it was registered, and sinus grafting was not done as well as implant placement. If perforation was not detected using extensive lighting and magnification, grafting and implant placement were done, then post‐placement radiographs were taken to second rule out any possible missed perforation. In addition, common sequelae following sinus membrane perforations, such as sinusitis, epistaxis, oroantral communication, nasal cavity penetration, exfoliation of graft particles from the nose, and maxillary ostium obstruction,^50^ were not observed in our treated patients, which reassured that clinically detectable membrane perforations were duly accounted for.

Although previous publications have reported the safety of the OD instrumentation in sinus membrane elevation, some of these are single case reports,^51^ whereas others include a reduced number of subjects, commonly less than 20.^40,44^ The lack of follow‐up of the restored implants and of the implant survival and success rates are the limitations of the present study, although its aim was to report the surgical approach and risk factors of SFE with OD in a multicenter setting. Although such data are not presented, all implants placed on the same day of the sinus grafting were restored and followed in routine‐maintenance recall, as mentioned in the results section, which suggests that implants osseointegrated and could be successfully restored. A previous multicenter study evaluated the up to 5‐year outcomes after 261 implant placements after SFE with OD (subsinus RBH of 5.4 mm resulting in a significant vertical increase of 7 mm), and it reported a 97% cumulative implant survival rate.^39^

It is important to note that not every sinus surgery received implant placement, because in case of perforation, the sinus case was counted as grafting case but with no implant placement associated with it due to perforation. Moreover, as observed with multicenter studies, one of their limitations are the need to work with a heterogeneous study population, different research team, yet investigating a common aim through the same methodology.^52^ These aspects may lead to slight discrepancies such as those observed in the present study for the number of implants and perforations among different study centers. On the other hand, the advantages of multicenter studies include a diverse and larger subject coverage, increased generalizability, and higher potential to provide robust information for future systematic reviews and meta‐analyses.^53^ Therefore, the present study aim was to examine sinus membrane perforations post‐OD instrumentation in 6 centers. The overall perforation rate was 7.31% in 670 sinus sites in 621 patients with 621 implant placements. Despite current valuable insights and robust evidence for dental field, prospective clinical studies are still paramount to investigate the long‐term performance of implants and implant‐supported restorations placed in sinus grafted areas using the transcrestal approach associated with OD instrumentation.

## 5 CONCLUSION

In the current multicenter clinical study, the postulated alternative hypothesis that OD instrumentation used for TSFE would result in low membrane perforation rate was accepted. Challenging scenarios of severe posterior maxillary atrophy are presented as risk factors for increased rate of membrane perforation.

## AUTHOR CONTRIBUTIONS

Ziv Mazor: Conceptualization, Data curation, investigations, methodology, and writing. Joao Gaspar: Conceptualization, Data curation, investigations, methodology, and writing. Robert Silva: Conceptualization, methodology, Data curation, and writing – review and editing. Snjezana Pohl: Conceptualization, methodology, Data curation, and writing – review and editing. Yazad Gandhi: Data curation, investigations, methodology, and writing – review and editing. Salah Huwais: Conceptualization, data curation, investigations, methodology, and writing – review and editing. Edmara T. P. Bergamo: Conceptualization, formal analysis, investigations, methodology, and writing. Estevam A. Bonfante: Conceptualization, investigations, methodology, and writing – review and editing. Rodrigo Neiva: Conceptualization, data curation, investigations, methodology, and writing – review and editing.

## CONFLICT OF INTEREST STATEMENT

Dr Salah Huwais developed the novel osseodensification technique and invented the patented multi‐fluted densifying burs which were utilized. None of the remaining authors have any conflicts to declare related to this study.

## Data Availability

The data that support the findings of this study are available on request from the corresponding author. The data are not publicly available due to privacy or ethical restrictions.
